# Efficiency of natural substances to protect *Beauveria bassiana* conidia from UV radiation

**DOI:** 10.1002/ps.5209

**Published:** 2018-10-25

**Authors:** Deborah Kaiser, Sven Bacher, Laurent Mène‐Saffrané, Giselher Grabenweger

**Affiliations:** ^1^ Department of Plant Protection Agroscope Zurich Switzerland; ^2^ Department of Biology University of Fribourg Fribourg Switzerland

**Keywords:** *Beauveria bassiana*, UV protection, biocontrol, humic acid, formulation

## Abstract

**BACKGROUND:**

Solar radiation is assumed to be a major factor limiting the efficacy of entomopathogenic fungi used as biocontrol agents in open field applications. We evaluated 12 natural UV‐protective co‐formulants for their effect on the survival of UV‐exposed *Beauveria bassiana* spores on agar plates, colza leaf discs and in the field.

**RESULTS:**

Colony‐forming unit (CFU) counts of unformulated conidia on agar plates and leaf discs dropped to ≤ 50% after exposure to UV radiation. The highest UV protection was achieved with humic acid, which provided > 90% protection of UV‐B‐exposed conidia in laboratory experiments. In the field, 10% humic acid increased spore persistence up to 87% at 7 days after application. Sesame and colza oil also provided high UV protection in both assays (> 73% and > 70%, respectively).

**CONCLUSIONS:**

This study shows that it is possible to increase the persistence of *B. bassiana* spores under exposure to UV radiation by formulation with natural UV‐protective additives. UV protectants might, therefore, increase the efficacy of entomopathogenic fungi as biocontrol agents in open field applications. © 2018 The Authors. *Pest Management Science* published by John Wiley & Sons Ltd on behalf of Society of Chemical Industry.

## 1 INTRODUCTION

The entomopathogenic ascomycete *Beauveria bassiana* is an established biocontrol agent in greenhouses and is used as an active ingredient in over 50 plant‐protection products worldwide.^1^ Although the stable conditions in greenhouses facilitate use of this entomopathogenic fungus (EPF), open field applications, particularly against airborne insect pests, are often limited by adverse environmental conditions such as direct solar radiation, temperature fluctuations or drought stress.^2^, ^3^ *Beauveria bassiana* is not adapted to tolerate direct sunlight and its conidia are rapidly inactivated upon exposure to ultraviolet (UV) radiation.^4^, ^5^, ^6^ This is assumed to be one of the main factors limiting the persistence and efficacy of EPFs in the field.^7^, ^8^, ^9^ Several mechanisms are involved in the inactivation of conidia by UV radiation. For example, UV‐B radiation (UV radiation at wavelengths between 280 and 320 nm) causes direct damage to DNA as strand breaks and base lesions that may lead to mutations or failures of transcription.^10^ Radiation at wavelengths between 320 and 400 nm (UV‐A) causes indirect damage by the production of radicals, which again have the potential to damage DNA. Thus, any effective use of *B. bassiana* as a microbial control agent under open field conditions, e.g. against airborne insect pests in arable crops, requires the development of a formulation that allows conidia to extend their survival on the phylloplane. Such a formulation must include co‐formulants that provide UV protection.

Similar formulations including UV protection for microbial control agents, such as viruses or bacteria, have been developed in several studies. For the biological control agent *Bacillus thuringiensis*, increased survival and efficacy under direct sunlight conditions was achieved by formulating a biological control agent with chemical UV absorbants and starch encapsulation.^11^, ^12^ For viruses, substances that showed UV protection include natural, organic compounds suchs as lignin, black and green tea, coffee and cocoa extracts, and other plant‐derived antioxidants.^13^, ^14^, ^15^, ^16^, ^17^, ^18^ However, only a few efficient and cost‐effective formulations have been commercialized to date.^19^

For EPFs, a number of substances showed excellent protection of conidia from sunlight inactivation in controlled environments, but reports of enhanced persistence in field settings are less common. Promising substances tested to date include optical brighteners, such as Tinopal, and natural compounds like clay, lignin and vegetable or mineral oils.^9^, ^20^, ^21^, ^22^, ^23^ Various oils helped to increase spore survival after UV‐B exposure in laboratory studies, but lost part of their protective potential in field assays or when applied on plant material.^20^, ^24^ Moreover, some formulations showed a UV‐protective effect, but seemed to interfere with the virulence of the EPF against the target host, e.g. lignin‐coated *B. bassiana* spores for control of *Lygus lineolaris*.^22^ Significant UV protection of *B. bassiana* conidia under field conditions was demonstrated by Inglis *et al*.^20^ using clay, a natural sun blocker, and Tinopal, an optical brightener, achieving a photoprotection of 25% and 37%, respectively.

However, the current choice of substances available as co‐formulants for effective UV protection of EPFs remains very limited. This constitutes a major drawback in the use of EPFs against airborne pests in open field applications.

The aim of this study was to test the potential of UV‐protective substances to enhance the survival of *B. bassiana* conidia in open field applications. In a previous study, *B. bassiana* isolates showed promising results as microbial control agents of pollen beetles (*Meligethes* spp.) in colza (*Brassica napus*).^25^ Because application of the microbial control agent is envisaged for all types of farming systems, we limited the choice of potential co‐formulants to natural substances, which may also be acceptable as adjuvants in organic colza production. In a first step, we tested several candidate natural UV protectants for their compatibility with *B. bassiana* conidia. We then evaluated their potential to protect conidia from UV‐B radiation in the laboratory. Finally, we assessed survival of conidia with and without the most promising formulation in the field.

## 2 MATERIAL AND METHODS

### 2.1 Fungus strain and conidia production

*Beauveria bassiana* strain ART2587 originates from a mycosed pollen beetle (*Meligethes* sp.) collected in Zurich, Switzerland.^25^ Single‐spore isolates of the strain are stored at −70 °C in 10% skim milk (Difco, Becton Dickinson, Franklin Lakes, NJ, USA). For use in experiments, the strain was grown on plates with 20 mL Sabouraud agar, modified, supplemented with 1% yeast extract (SDAY; Difco, Becton Dickinson), and incubated in the dark at 22 °C and 75% relative humidity (RH) for 2 weeks. Conidia were collected by rinsing plates with 0.1% Tween 80.

### 2.2 Selection and formulation of UV‐protective additives

Selection of natural substances with potential UV‐protective effects was based on previous studies with a focus on water‐soluble additives.^14^, ^15^, ^17^, ^20^, ^22^, ^26^, ^27^, ^28^, ^29^, ^30^ In addition, substances were included that are already used in organic colza cultivation as plant growth stimulants, wetting agents or adhesives for insecticides (Table 1).

**Table 1:** Substances evaluated for their UV‐protective potential of *Beauveria bassiana* conidia

Substance	Ingredients	Manufacturer/distributor
Humic acid, potassium	78% humic acid, 0.45% potassium	WH Pharmawerk Weinboehla GmbH, Weinboehla, Germany
Humic acid, sodium	65% humic acid, 7.2% sodium	WH Pharmawerk Weinboehla GmbH, Weinboehla, Germany
*Reseda luteola* extract	62.5% polyphenols (luteolin, apigenin), 2% ash, 4% water	NIG Nahrungs‐Ingenieurtechnik GmbH, Magdeburg, Germany
*Hippophae rhamnoides* extract	55% polyphenols (ellagitannins, flavonoids), 9.8% proteins, 3% water, 15% titratable acid, 7% ash, 0.3% fat	NIG Nahrungs‐Ingenieurtechnik GmbH, Magdeburg, Germany
Lignin, alkali	Lignin	Sigma‐Aldrich, St Louis, MO, USA
Yeast extract	Yeast extract	Merck Millipore, Darmstadt, Germany
Skim milk	Skim milk	Difco, Becton Dickinson, Franklin Lakes, NJ, USA
Surround®	95% kaolin	Staehler Suisse SA, Zofingen, Switzerland
KlinoSpray (zeolite)	70% silica, 2.8% potassium oxide, 2.5% calcium	Unipoint AG, Ossingen, Switzerland
Black tea	143 000 mg kg^−1^ catechin, 109 000 mg kg^−1^ epicatechin, 179 000 mg kg^−1^ gallic acid, 216 000 mg kg^−1^ tannic acid	Tee Gschwender, Meckenheim, Germany
Green tea	106 000 mg kg^−1^ catechin, 80 700 mg kg^−1^ epicatechin, 133 000 mg kg^−1^ gallic acid, 160 000 mg kg^−1^ tannic acid	Twining and Company Limited, London, UK
Colza oil	274.4 ng μL^−1^ alpha‐tocopherol, 7.06% saturated fatty acids, 0.06% free fatty acids, 0.04% myristic acid, 64.5% oleic acid, 18.3% linoleic acid	Omya AG, Oftringen, Switzerland
Sesame oil	78.7 ng μL^−1^ alpha‐tocopherol, 15.8% saturated fatty acids, 0.7% free fatty acids, 39.6% oleic acid, 40.8% linoleic acid	Migros‐Genossenschafts‐Bund, Zürich, Switzerland

Liquid stock suspensions of powdery substances were freshly prepared for each assay in 0.1% Tween 80 (180 mg to 1320 μL 0.1% Tween 80). Tea was brewed in sterile distilled water at 70 °C for 30 min (2.2 g bulk tea to 17.8 mL distilled water) and the liquid portion complemented with Tween 80 to obtain a stock solution containing 0.1% Tween 80. Pure oils were mixed with 15% v/v Tween 80 as emulsifier. For the assays, stock solutions were added to spore suspensions to obtain 10% v/v of the UV protectant.

### 2.3 Compatibility of *B. bassiana* with UV‐protective additives on agar plates

The number of colony forming units (CFUs) of *B. bassiana* growing on plates with and without UV‐protective substances was used as a measure of the compatibility of the fungus with the tested co‐formulants. Conidia (1 × 10^3^ conidia mL^−1^) suspended in 0.1% Tween 80 were complemented with 10% v/v of test substance, thoroughly mixed and 100 μL plated with sterile glass beads onto SDAY plates. Conidia suspended in 0.1% Tween 80 only served as a control. A variant with 1.7% v/v Tween 80 was included because it is an emulsifier in the tested oil variants. Agar plates were incubated in the dark at 22 °C and 75% RH and CFUs counted after 5 days. All substances were tested in three independent runs, each with three replicates.

### 2.4 UV‐B protection on agar plates

The detrimental effects of UV exposure on conidia seem to be mainly caused by UV‐B radiation.^9^, ^31^, ^32^, ^33^ This fraction of the solar spectrum is therefore used to evaluate UV‐protective substances and the tolerance of fungus isolates to solar exposure.^6^, ^24^, ^34^

SDAY plates with conidia suspensions were prepared as described above, and incubated for 30 min without light prior to UV exposure. After that, plates were transferred to a climate chamber (Climecab 1400, Kälte 3000 AG, Switzerland) onto shelves at three levels. Each level was equipped with polychromatic SolarRaptor T5 UV‐B (Econlux GmbH, Cologne, Germany) strip‐lights at a distance of 25 cm from the exposed plates.

Strip‐lights emitted an average UV‐B irradiance of 2792 mW m^−2^ to agar plates at this distance, which was measured with a manual solarmeter model 6 (Solar Light Co., Glenside, PA, USA). Plates were incubated for 4 h at 22 °C and 75% RH, resulting in a total UV‐B dose of 40.3 kJ m^−2^. This corresponds to the cumulative UV‐B dose during 2 days in lowland Switzerland in mid‐April, the point when treatments against pollen beetles in colza are usually carried out. To get this UV‐B dose, UV erythema measurements of the weather station in Payerne, Switzerland, provided by Meteo Swiss (Zurich, Switzerland), were used. Using a tropospheric ultraviolet and visible radiation model (TUV) calculator (http://cprm.acom.ucar.edu/Models/TUV/Interactive_TUV) the average ratios of UV erythema and UV‐B during the course of a day were calculated and applied to the real UV erythema measurements.

Four plates for each treatment were exposed to UV‐B irradiance at the same time. To ensure equal irradiance of test samples, agar plates were placed on a rotating disc 25 cm in diameter, mounted on shelves beneath the strip‐lights. Another three plates with the same treatments were placed in a separate part of the climate chamber, which was protected from UV radiation by aluminum foil shields. After incubation, agar plates were covered with lids, transferred to another climate chamber and kept in the dark at 22 °C and 75% RH for 5 days. Percentage survival of UV‐B radiated conidia for each variant was calculated as proportion of CFUs of irradiated and non‐irradiated plates of the same treatment within each experiment. To avoid potential influence of plate border reflection on CFU formation, only CFUs from the central circular surface of each plate with a diameter of 5 cm were included in the analysis. Each substance was tested in three independent runs, once on each shelf level of the climate chamber.

### 2.5 UV‐B protection on colza leaf discs

Leaf discs of greenhouse‐grown colza were surface‐sterilized in 70% ethanol for 30 s, followed by 1 min in 1% chloramine‐T trihydrate and subsequently rinsed in sterile distilled water (adapted from Vidal *et al*.^35^). One replicate consisted of three leaf discs of 1 cm diameter placed in the center of a 2% water agar plate, 55 mm in diameter. Three 1‐μL droplets of conidia suspension (3 × 10^5^ conidia mL^−1^ suspended in 0.1% Tween 80 and mixed with 10% v/v test substance) were pipetted onto the upper surface of each leaf disc. Conidia suspended in 0.1% Tween 80 served as a negative control. A treatment with 1.7% v/v Tween 80 as a test substance was included because this amount is used as an emulsifier in the tested oil variants. Droplets were left to air‐dry for 30 min. Afterwards, two plates of each treatment were exposed to UV‐B radiation as described above. UV radiation time was 5 h with a total UV‐B dose of 49.3 kJ m^−2^. This is the average UV‐B irradiance measured over 2.5 days in lowland Switzerland mid of April. Another two plates of each treatment were incubated in a separate, aluminum foil‐shielded part of the same climate chamber. Only one shelf level of the climate chamber was used in leaf disc experiments.

After UV‐B radiation, all three leaf discs of one plate were placed in 1 mL of 10 mm phosphate‐buffered saline (PBS) with 0.05% Tween 20 (pH 7.4) and homogenized with tungsten carbide beads in a TissueLyser (Qiagen, Hilden, Germany). One hundred microliters of the homogenate was spread onto each of three SDAY plates and incubated in the dark at 22 °C and 75% RH for 7 days before CFUs were counted. Percentage survival of UV‐B radiated conidia for each variant was calculated as proportion of CFUs found on irradiated and non‐irradiated plates of the same treatment. Each substance was tested in three independent runs, each with two replicates.

### 2.6 Field experiment

Based on the results of the above‐described laboratory trials, humic acid sodium was tested as a UV‐protective co‐formulant in a field trial in 2017, consisting of three treatments: (i) 1 × 10^13^ ha^−1^ *B. bassiana* conidia in 0.1% Tween 80, (ii) 1 × 10^13^ ha^−1^ *B. bassiana* conidia in 0.1% Tween 80 and 10% humic acid sodium, and (iii) an untreated control. *B. bassiana* conidia were produced on sterile barley kernels by Mycelia GmbH (Nevele, Belgium), and harvested with a MycoHarvester (MH5, VBS Agriculture Ltd, Beaconsfield, UK). The unformulated powder contained 1.3 × 10^11^ conidia/g with >70% viability. The colza field (variety Avatar) was situated in eastern Switzerland (Taenikon, 47°28′46″N, 8°54′26″E). It was divided into plots of 285 m^2^ (15 × 19 m) with six plots per treatment arranged randomly in a rectangle. Test substances were applied at a rate of 600 L ha^−1^ (17.1 L plot^−1^) using a machine mounted 15 m spray bar (Fischer AG, Schenkon, Switzerland) equipped with 30 nozzles AIC 11005 (TeeJet, Wheaton, IL, USA) on 6 April 2017. Meteorological parameters including total solar radiation, temperature and precipitation were recorded by a local weather station.

Survival of fungus spores on colza was assessed on days 3, 7 and 14 after application. At each sampling date, the main inflorescences of five randomly selected colza plants were collected in the center of each plot, brought to the laboratory and stored at 6 °C for a maximum of 7 days. Buds were separated from stems with sterilized scissors and transferred to an extraction bag (Bioreba, Reinach, Switzerland). Bags were filled with 15 mL of 0.01 m PBS containing 0.05% Tween 20 and the plant material was homogenized using an electronic homogenizer (Bioreba). One hundred microliters of the homogenate was plated in triplicate on Sabouraud 2% glucose agar (SDA) containing antibiotics (cycloheximide, 0.05 g L^−1^; streptomycinsulfate, 0.6 g L^−1^; tetracycline, 0.05 g L^−1^) and the fungicide dodine (50 mg L^−1^).^36^ CFUs were counted after incubation at 22 °C and 75% RH for 14 days and storage at 6 °C until evaluation. To get the average number of CFUs per main inflorescence, the CFUs per total plant homogenate was calculated and divided by five.

### 2.7 Statistical analyses

Linear mixed effect models were used for statistical evaluation. They were fitted by REML using the package ‘nlme’ (version 3.1‐128; Pinheiro *et al*. 2017)^37^ in the statistical software R (version 3.2.3; R Development Core Team, 2015).

For compatibility tests of *B. bassiana* spores with UV‐protective substances, the number of CFUs was chosen as the response variable, which was log‐transformed to meet the assumption of normally distributed residuals. The UV‐protective substance tested was the explanatory variable. The replicate numbers nested within the single runs were included as random factors.

To analyze *B. bassiana* conidia survival after UV‐B exposure on agar plates and on colza leaf discs, the CFU count per plate was the response variable, which was log‐transformed to meet the assumption of normally distributed residuals. To determine whether a UV‐protective substance had a positive effect on spore survival upon UV‐B exposure, relative to the spore survival of the untreated variant, the interactions of the UV‐protective substances tested and the treatment (UV exposure yes/no) were included as explanatory variables. The replicate number nested within the experiment was included as a random factor. For the agar plate assay, the shelf level in the climate chamber nested within each single run was also included as random factor.

The effect of humic acid sodium on the survival of *B. bassiana* conidia in the field experiment was analyzed with the CFU counts per main inflorescence as the response variable, which was log‐transformed to meet the assumption of normally distributed residuals. The tested treatment was the explanatory variable and the plot number was included as a random factor.

## 3 RESULTS

### 3.1 Compatibility of *B. bassiana* conidia with UV‐protective additives

All substances tested, except for the *H. rhamnoides* extract, showed good compatibility with no significant inhibition of *B. bassiana* conidia. The *H. rhamnoides* extract significantly reduced (*P* < 0.001) *B. bassiana* CFU formation by 97.2 ± 5.8% (mean ± SD). Compatibilities of the remaining substances ranged from 82% to 109%, with black tea and sesame oil showing the lowest compatibilities of 82.1 ± 21.8% and 83.7 ± 35.7%, respectively. Humic acid sodium and the zeolite showed best compatibilities with 109 ± 38.1% and 106 ± 37.7%, respectively. All substances except for the *H. rhamnoides* extract were further evaluated for their UV‐protective potential.

### 3.2 UV‐B protection on agar plates

**Figure 1:**
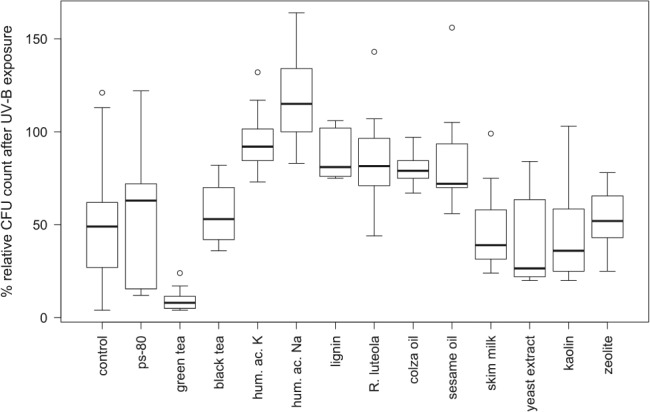
Relative colony‐forming unit (CFU) count of *Beauveria bassiana* conidia on agar plates after exposure to UV‐B radiation for 4 h. Test substances were used at a 10% concentration. Polysorbate 80 was tested at a 1.77% concentration because it was used as an emulsifier for oil additives. Percentage survival was calculated in relation to non‐irradiated plates within each experiment. hum. ac. K, humic acid potassium; hum. ac. Na, humic acid sodium; ps‐80, polysorbate 80; *R. luteola*, *Reseda luteola*.

Four hours of exposure of UV‐B radiation led to a clear reduction in viable conidia in the unformulated control, with a relative CFU count (CFU of irradiated plates/CFU of non‐irradiated plates) of 48.1 ± 23.3% (Fig. 1). Of the 12 additives tested, six showed a clear UV‐protective effect with significantly higher conidia counts after irradiation compared with unformulated conidia (*P* < 0.05; Fig. 1 and Table 2). The highest UV‐B protection was achieved by the addition of humic acid sodium, resulting in a relative conidia count of 112.6 ± 14.0%. Treatments containing black tea, colza oil, sesame oil, luteolin and humic acid potassium also showed significantly increased CFU counts compared with controls, resulting in relative CFU counts of 53.8–94.7%. Conidia formulated with green tea exhibited significantly lower CFU counts after UV‐B exposure compared with the control. An adverse effect of green tea on conidia survival was, however, not detected in non‐irradiated plates. The same was true for all the other substances tested, confirming the compatibility results.

**Table 2:** UV‐protective effect of tested substances on *Beauveria bassiana* conidia after UV‐B exposure

	Agar assay					Leaf disc assay				
Substance	Estimate	SE	df	*t*‐value	*P*‐value	Estimate	SE	df	*t*‐value	*P*‐value
Polysorbate 80	0.021	0.08	52	0.263	0.790	0.325	0.09	148	3.535	0.001
Green tea	−0.277	0.08	52	−3.342	0.002	0.210	0.09	148	2.423	0.017
Black tea	0.294	0.08	52	3.597	0.001	0.070	0.09	148	0.771	0.441
Humic acid K	0.221	0.08	52	2.751	0.008	0.732	0.09	148	7.834	0.000
Humic acid Na	0.301	0.08	52	3.850	0.000	0.689	0.09	148	7.581	0.000
Lignin	0.145	0.08	52	1.799	0.078	0.645	0.09	148	7.118	0.000
*R. luteola*	0.305	0.08	52	3.742	0.001	−0.198	0.09	148	−2.187	0.030
Colza oil	0.177	0.08	52	2.198	0.032	0.580	0.09	148	6.371	0.000
Sesame oil	0.173	0.08	52	2.197	0.033	0.567	0.09	148	6.281	0.000
Skim milk	−0.045	0.08	52	−0.574	0.569	0.596	0.09	148	6.618	0.000
Yeast extract	−0.018	0.08	52	−0.225	0.823	0.435	0.09	148	4.825	0.000
Kaolin	0.068	0.08	52	0.857	0.400	0.497	0.09	148	5.500	0.000
Zeolite	−0.112	0.08	52	−1.381	0.172	0.270	0.09	148	2.981	0.003

A linear mixed effect model was built using logarithmic colony‐forming unit (CFU) counts as the response variable and the interactions of the UV‐protective substances tested and the treatment (UV exposure yes/no) as explanatory variables. Only the statistics of the interaction terms (compared with the unprotected control) are displayed. The UV‐protective effect of a substance is indicated by a significant interaction term.

### 3.3 UV‐B protection on colza leaf discs

**Figure 2:**
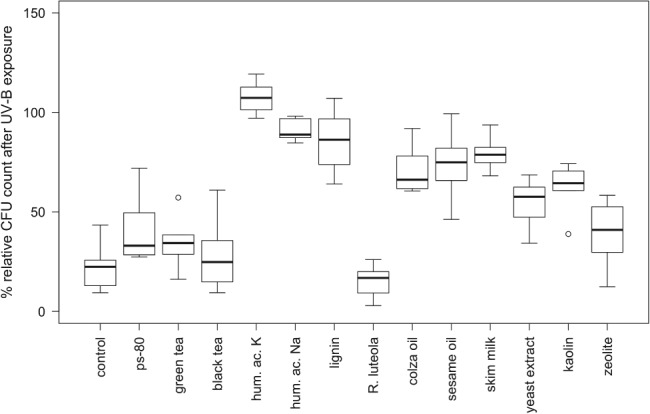
Relative colony‐forming unit (CFU) count of *Beauveria bassiana* conidia on colza leaf discs after exposure to UV‐B radiation for 5 h. Test substances were used at a 10% concentration. Polysorbate 80 was tested at a 1.77% concentration because it was used as an emulsifier for oil additives. Percentage survival was calculated in relation to non‐irradiated plates within each experiment. hum. ac. K, humic acid potassium; hum. ac. Na, humic acid sodium; ps‐80, polysorbate 80; *R. luteola*, *Reseda luteola*.

After 5 h of UV‐B radiation, the relative CFU count of unformulated conidia was reduced to 21.7 ± 9.2% (Fig. 2). All compounds tested, except for black tea and *R. luteola* extract, caused a significant reduction in conidial mortality from UV‐B exposure (*P* > 0.05, Fig. 2, Table 2). The best UV‐B protection was provided by humic acid potassium with a mean relative CFU count of 111.9 ± 46.6%, followed by humic acid sodium and lignin (Fig. 2). The addition of *R. luteola* extract caused a significant decrease in the survival of conidia exposed to UV‐B (Fig. 2, Table 2). CFU counts in non‐irradiated plates were similar for all substances tested, with the exception of green tea, which exhibited a significantly lower CFU formation relative to the control plates (*P* = 0.049).

### 3.4 Field experiment

The first 3 days after application were cloudless with a total solar radiation of 7 × 10^4^ kJ m^−2^. Based on the estimation that 5% of the total solar radiation is UV radiation and 5% of that is UV‐B radiation^38^, the UV‐B dose in the field trial exceeded the dose applied in laboratory trials after 1 day.

Subsequent days were more variable with total solar radiation of 7.9 × 10^4^ and 8.1 10^4^ kJ m^−2^ during the second and third periods between sampling dates, respectively. Whereas the first 8 days were rainless, precipitation was recorded for 5 days from day 9 after application (27 mm in total).

**Figure 3:**
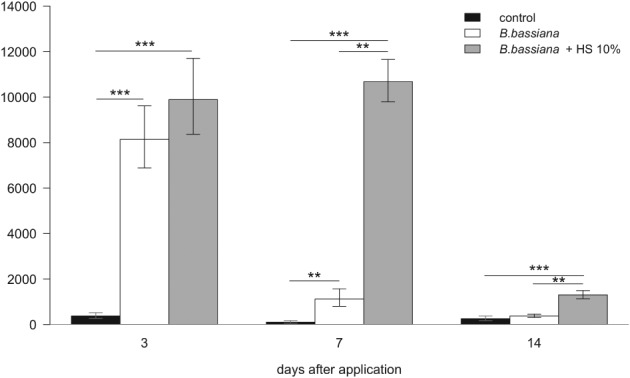
Number of *Beauveria bassiana* colony‐forming units (CFUs) per main colza inflorescence at days 3, 7 and 14 after application. Treated variants include *B. bassiana* conidia with and without 10% humic acid sodium. *B. bassiana* CFUs from main inflorescences of untreated plots served as the control. Standard errors are based on back‐transformed logarithmic values of six replicates. Asterisks designate significant differences between treatments: *P < 0.05, **P < 0.01, and ***P < 0.001.

CFU counts in fungus treatments were significantly higher on day 3 than in the untreated control. However, they were not significantly different between fungus treatments (Fig. 3). CFU counts dropped substantially between day 3 and day 7 in the fungus treatment without UV protectant, whereas they remained almost unchanged in the treatment with the UV protectant included. Significantly more conidia survived until day 7 when formulated with the UV protectant humic acid sodium with 7.8 times higher CFU counts on colza main inflorescences than in the unformulated variant. Comparison at day 14 revealed a further loss in viable conidia in both formulations. However, the difference in conidia survival was still significant between treatments, with three times more CFUs growing in samples from the treatment including the UV protectant. By contrast, CFU counts of the fungus treatment without UV protectant reached the same low levels as the untreated control.

## 4 DISCUSSION

UV‐B radiation caused a significant drop in survival of conidia in our laboratory and field trials if exposed without UV‐protective additives. This is in line with previous studies showing the susceptibility of *B. bassiana* conidia to UV radiation.^5^, ^6^ Our study has shown that it is possible to increase the persistence of *B. bassiana* spores under exposure to UV radiation by formulation with natural UV‐protective additives. Among our tested substances we identified humic acid as the most promising additive with > 90% UV‐B protection in laboratory experiments, in contrast to a significant reduction in CFU counts of unformulated conidia of 50% or more. Moreover, under realistic field conditions, we achieved a significant increase in spore survival in the field, 7 and 14 days after application. To the best of our knowledge, this is the first time that humic acid has been tested as a UV protectant in a formulation with an entomopathogenic fungus, although its potential has been indicated in previous studies. Bitton *et al*.^26^ and Muela *et al*.^27^ tested humic acid successfully as a UV protectant of bacteria, and John^39^ was able to protect a human cell line from UV deactivation using humic acid. Its UV‐protective effect seems to be based mainly on the specific absorption of UV radiation by organic molecules.

A similar UV‐absorbing capacity of the organic molecule may also explain the efficacy of lignin as a UV‐protective co‐formulant, as shown in our experiments. In a previous study, a formulation with cross‐linked lignin‐coated *B. bassiana* conidia also showed high UV protection under simulated sunlight.^22^ However, this formulation also showed reduced insecticidal effects of the fungal spores against its target insect, the bug *Lygus lineolaris*.

Some vegetable and mineral oils have shown excellent UV‐absorbing properties as well. Significant UV protection has been shown when conidia of *B. bassiana*, suspended in oils were spread on glass slides under controlled conditions. The same oils, however, did not enhance spore survival in field trials. One reason for this loss of UV protection of oils on plant foliage is their possible absorption into the mesophyll cells of plant leaves.^20^, ^24^ In our experiments, however, we observed high UV protection with vegetable oils such as sesame and colza oil spread on agar plates, and similar levels of protection on colza leaf discs. The waxy upper leave surface of colza (and brassicas in general) may have prevented absorption of the oils into the leaf mesophyll. However, it remains questionable whether the waxy, and therefore lipophilic, layer on the colza leaf surface is able to prevent absorption of other lipophilic substances.

Contrary to what we found with vegetable oils, some of the substances tested showed inconsistent results on the two experimental systems used, agar plates and leaf discs. For example, the two stone meals tested as UV blockers, kaolin and zeolite, both showed a UV‐B protective effect only when spread on leaf discs. One explanation may be that the solid stone meal particles, which are responsible for blocking UV radiance, were absorbed by the medium in our agar plate assay and therefore no longer able to shield the conidia. This was probably not the case on leaf discs, where the wax layer on the leaf surface possibly prevented absorption and UV‐blocking particles stayed on the surface. The natural UV blocker clay has already been tested previously in field trials by Inglis *et al*.^20^ showing an average photoprotection of *B. bassiana* conidia on crested wheatgrass of 25–37%.^40^ Similarly, kaolin, a clay mineral, has been shown to provide moderate protection against UV radiation of *Cydia pomonella* granulosis virus in laboratory studies.^16^

The opposite effect, significant UV protection on agar plates and a strong decrease in efficacy on leaf discs, was found with the application of *R. luteola* extract as a co‐formulant. *Reseda luteola* plants contain high amounts of the flavonoid luteolin which is able to absorb UV‐A and UV‐B radiation. It is also known to have antimicrobial properties towards bacteria, yeast and fungi.^30^, ^41^, ^42^ One reason for the contradictory results achieved with *R. luteola* extract may therefore be a toxic side‐effect of the extract on EPF spores. We did, however, not detect any evidence of such an effect in our initial compatibility test. Evaporation of the spore suspension may, however, have led to an increase in the concentration of luteolin on treated leaf discs and, consequently, to a decrease in spore survival caused by the antimicrobial properties of the higher concentrated co‐formulant. However, the agar assay revealed more variable results than the leaf disc assay and should therefore be more carefully interpreted.

Green and black tea have shown high UV protection in entomopathogenic viruses in previous studies, whereas we did not find a similar strong effect for *B. bassiana* conidia in our study.^14^, ^17^, ^18^ Green and black tea contain high amounts of polyphenols. These act as antioxidants for radicals triggered by UV radiation but show very little UV‐A or UV‐B absorption.^43^ It has been shown that the UV protection of viruses and bacteria is in part attained by antioxidants and antioxidative enzymes, which are able to counteract the damage caused by radicals at the site of the DNA.^44^ We assume that polyphenolic compounds of green or black tea may more easily enter bacterial cells or viral bodies, whereas they may fail to get through the multilayered wall of the resting *Beauveria* conidia. As a consequence, they would not be able to exhibit their antioxidative activity in the latter. It seems reasonable that UV protection in *B. bassiana* conidia is given mainly by the UV‐blocking or absorbing capacities of additives, which act as a shield on the outside of spores.

The only substance that caused strong inhibition of *B. bassiana* CFU formation in our study was the *H. rhamnoides* extract. Its antifungal properties might be based on its high ascorbic acid content because this vitamin has been shown to have fungistatic potential towards a dermatophyte.^45^ Although not successful for purposes of this study, *H. rhamnoides* extract might be an interesting candidate for any types of treatments in which antifungal properties are desired, e.g. treatment of fungal plant pathogens, or even in human medicine.

Our results show that the formulation of EPF spores with UV‐protective substances can prolong the persistence of EPF spores exposed to UV radiation in the laboratory and in the field for several days. We assume that this is an important step towards enhancing the efficacy of EPF treatments (and treatments with any microbial control agent in general), because efficacy should increase with the length of time that target insects are exposed to viable infective spores.

Recent studies indicate that a secondary pickup of EPF spores from the foliage may indeed be as important for pest control as hitting target insects directly during spray application. Behle *et al*. demonstrated that *B. bassiana* residues on leaves may be as effective against a lepidopteran pest as direct spray treatments.^46^ *Metarhizium anisopliae* or *B. bassiana* residual spores on host plants of locusts or colorado potato beetles, respectively, caused a significant reduction in pest abundance, sometimes as high as in direct spray treatments.^47^, ^48^, ^49^, ^50^ However, such increase in efficacy strongly depends on the mobility and physical contact between the target insect and its host plant. Also, the feeding behaviour influences spore acquisition and thereby the route and probablity of infection. In any case, the efficacy of an EPF treatment depends on the number of infectious propagules that touch the insect cuticle, no matter if by primary or secondary pickup (see Jaronski^40^).

Declining pest mortalities observed in the field after application were attributed to the loss of viable spores due to adverse environmental factors, such as solar radiation.^40^, ^46^ Hence, the development of formulations to increase persistence of EPF spores under exposure to solar radiation is probably a keystone in the successful use of EPFs as biological control agents in field crops. Humic acids have proven high UV protection potential in our study, and may be an important additive to such a formulation. Field experiments in colza with a combination of *B. bassiana* spores and humic acid will show if the biocontrol control measure leads to a significant reduction of pollen beetle abundance. If so, the developed formulation would be a further valuable tool to foster the sustainable above‐ground control of pest insects in arable crops.
